# Osteosynthesis of a Multifragment Femoral Shaft Fracture and Peri-Implant Refracture in an 83-Year-Old Patient with Osteogenesis Imperfecta

**DOI:** 10.1155/2020/8887644

**Published:** 2020-07-13

**Authors:** Tobias M. Ballhause, Roland Gessler, Matthias H. Priemel, Karl-Heinz Frosch, Carsten W. Schlickewei

**Affiliations:** Department of Trauma and Orthopaedic Surgery, University Medical Center Hamburg-Eppendorf, Hamburg, Germany

## Abstract

*Introduction*. Osteogenesis imperfecta (OI) is the term for a heterogenic group of conatal diseases that affect the bone formation. Eight different OI types are known. Patients with types III and IV frequently suffer from fractures without adequate trauma. The literature gives plenty advice for fracture treatment in pediatric OI patients, but there is less for adults, and no recommendations can be found for geriatric OI patients. *Case Presentation*. We report on an 83-year-old male who suffered from OI type IV. He was able to walk with an individually adapted gait orthosis. In an accident, the patient sustained a distal, multifragment, femoral shaft fracture. The fracture was openly reduced and fixated with a retrograde inserted elastic stable intramedullary nail (ESIN). Three months later, the patient was capable of walking without crutches. Due to another accident, he sustained a peri-implant refracture without failure of the ESIN. We immobilized the leg, and it achieved bony healing without reosteosynthesis. Eleven weeks later, he was again able to mobilize himself with full weight bearing. *Discussion*. We present a unique case of osteosynthesis in a distal, multifragment, femoral shaft fracture in a geriatric OI patient. No recommendations for the treatment of mature patients with OI can be found in the literature. We present our treatment concept and technique of osteosynthesis with an ESIN. Despite another accident with a peri-implant refracture, sufficient bony healing occurred, which allowed the patient to freely mobilize himself again.

## 1. Introduction

Osteogenesis imperfecta (OI) describes several heterogenetic conatal diseases of the tissue and bone. All these different diseases result in a distinctive phenotype with brittle bones, disorganized growth plates, and short stature [1]. This phenotype has been known for a long time. In the 1840s, Willem Vrolik introduced the term to describe a newborn with multiple fractures [2].

Modern molecular techniques have revealed different genetic origins of OI, leading to a numeration of the various forms according to the genetic. In total, 8 types of OI have been described in the literature [3]. The most common involves mutations in the genes COL1A1 and COL1A2. These two genes encode the alpha-1 and alpha-2 chains of collagen type 1. Mutations in these genes are responsible for 85% of all cases of OI [4].

Most forms of OI are inherited autosomal-dominant [5]. A lag in collagen development results in skeletal dysplasia and a higher incidence of fractures, especially in type III and IV OI patients. So far, a causal therapy does not exist [6]. The treatment of fractures in patients with OI is challenging because of the altered bony morphology and density. Most plates and nails are too rigid and lead to new fractures or implant failures.

Over the last years, various distinctive implants have been developed, especially for children with OI. For example, the Fassier-Duval nail can be prophylactically implanted into long bones. Its telescopic characteristic allows the implant to lengthen as the child grows [7]. However, we could not find a treatment recommendation for femoral shaft fractures in geriatric patients with OI. We report such a case of geriatric OI patient. We found that the case was unique and decided to publicize our treatment and the result.

## 2. Case Presentation

We report an 83-year-old Caucasian male with OI type IV. The patient fell into a track bed with his electronic wheelchair. After recovery and transportation to the emergency department, a femoral shaft fracture was diagnosed. Although the hospital was a level-1 trauma center, they contacted our university medical center due to the rareness of the case.

**Figure 1:**
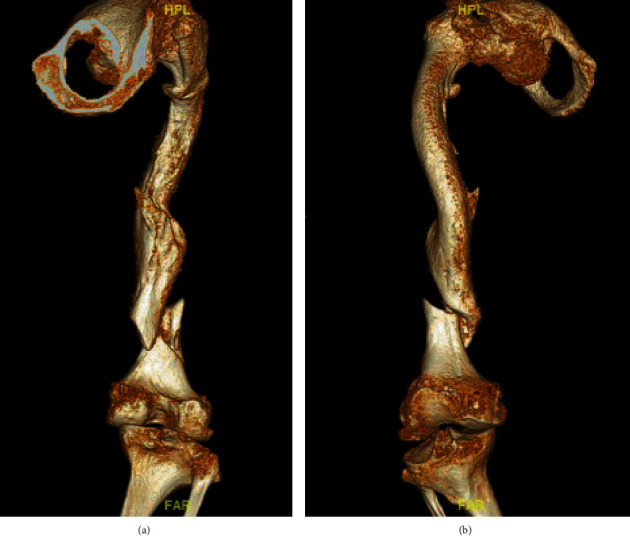
3D computed tomography of the right femur after admission to our emergency department. The images show the fractured femur on the second day after an accident. (a) Femur from dorsal view, where the multifragment characteristic of the fracture can already be assumed. (b) Corresponding ventral view of the femur.

The patient was transferred to us via helicopter. After arrival at our emergency department, we performed 3D computed tomography to better understand the morphology of the fracture (Figure 1). Due to the underlying disease and the bone structure in the 3D CT, we decided that a sufficient elastic osteosynthesis would be necessary to prevent further fractures or implant failure.

Matters were made worse by the patient's obesity (BMI: 74.4). Besides OI, he also suffered from the following comorbidities: constructive pulmonary disease (COPD), obstructive sleep apnea syndrome (OSAS), and arterial hypertonia. Surgery was performed 5 days after the accident and 2 days after the interhospital transfer of the patient. The patient was placed in a supine position, and the fracture site was prepared using a lateral approach.

**Figure 2:**
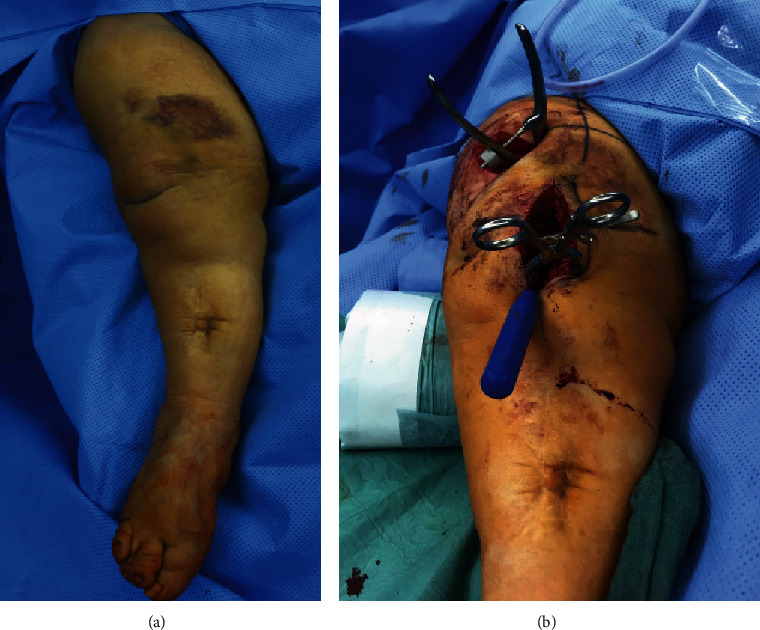
Clinical picture of the fractured femur in the operating room (a). Intraoperative picture of the situs after repositioning of the fracture (b).

**Figure 3:**
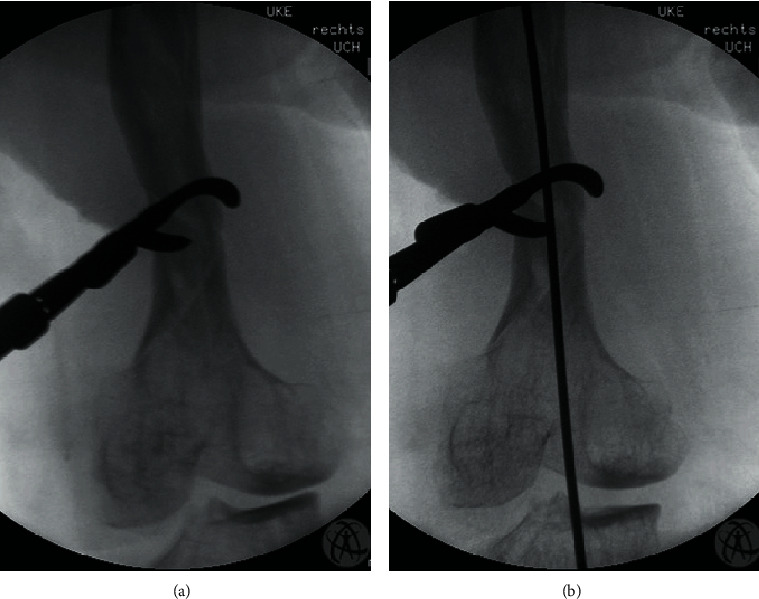
Intraoperative C-arm images. (a) Three major fragments after reduction temporarily fixated with a sharp reduction clamp. (b) Retrograde implantation of the ESIN.

The alignment of the femur was reconstructed by careful manual extension on the leg (Figure 2). Then, the three parts of the fracture were reduced with sharp reduction forceps (Figure 3(a)). When sufficient reduction was achieved, a 3.5 mm elastic stable intramedullary nail (ESIN) was carefully inserted from a retrograde position (Figure 3(b)). As the ESIN was placed, the reduction forceps were removed, and the osteosynthesis proved to be stable. The wound was closed, and the leg was elastically wrapped. Additionally, the thigh was immobilized with a cast for one week.

**Figure 4:**
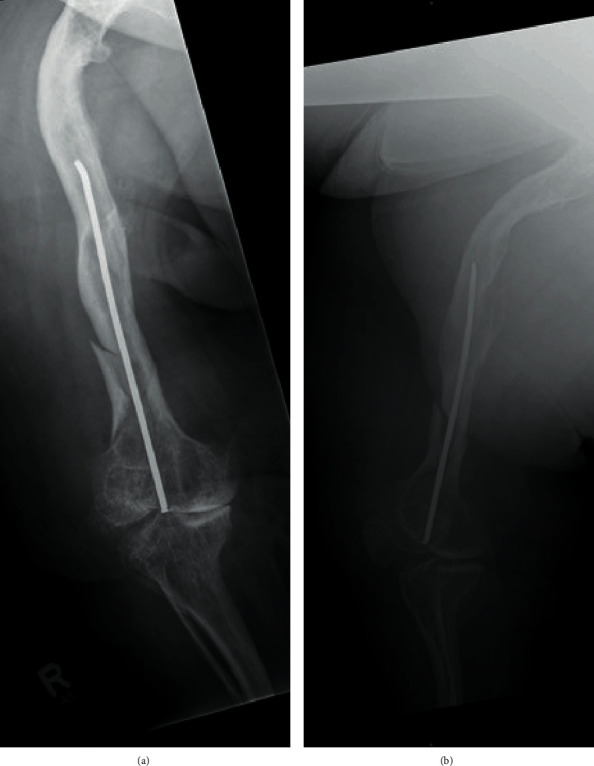
Postoperative radiographs from the second day after surgery. (a) Operated femur in the anteroposterior plane. (b) Corresponding lateral plane. A near-anatomical reduction of the femur has been achieved.

A postoperative weight-bearing restriction was imposed for six weeks. The patient received physiotherapeutic training after the cast was removed. Moreover, pain medication and thrombosis prophylaxis were prescribed. Eight days after surgery, the patient was released from hospital. The radiograph showed sufficient bony healing six weeks after the osteosynthesis (Figure 4). Three months after the accident, the patient was able to walk without further support aside from his gait orthoses.

**Figure 5:**
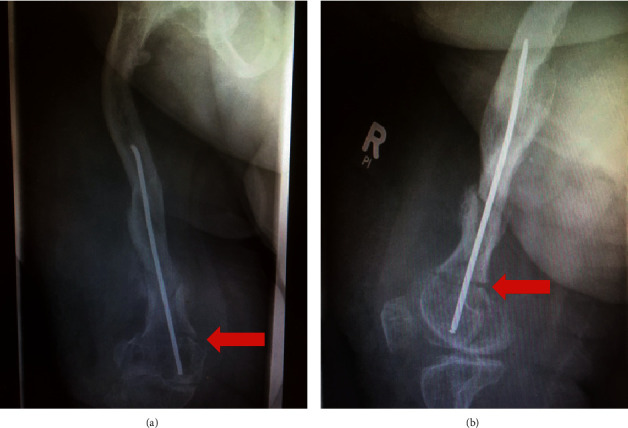
Peri-implant refracture four months after the initial surgery. (a) Anteroposterior view. The red arrow indicated the fracture. It is only minorly dislocated. (b) The corresponding lateral view shows the fracture and the unchanged position of the ESIN.

One month later, the patient fell again when he tried to mobilize himself. He came to our emergency department with the help of his wife, and a peri-implant fracture was diagnosed without failure of the ESIN (Figure 5). Closer analysis of the fracture showed almost no dislocation, and the elastic nail was stable. Thus, we recommended a conservative treatment with weight-bearing limitation for 6 weeks and immobilization with a stiff orthosis.

**Figure 6:**
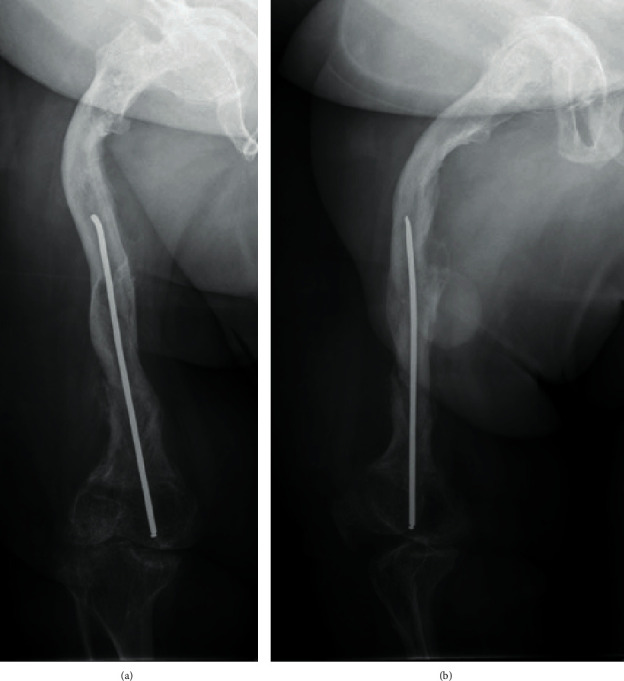
Bony healing 6 weeks after conservative treatment of the peri-implant fracture. (a) Anteroposterior view. The fracture is almost not visible anymore. (b) Lateral view. Callus tissue indicates the healing process of the bone. A gap can be seen, so we developed a scheme for the gradual increase of weight bearing.

The patient was treated in our outpatient clinic, and the radiograph showed sufficient bony healing six weeks after the refracture (Figure 6). A weight-bearing restriction of 15 kg for one more week was imposed. Gradually, the weight bearing was increased 15 kg each week. After 6 weeks, the patient reached full weight bearing. Thus, 12 weeks after the second accident, he was able to walk again with his gait orthosis, but without crutches. Seven months after the second accident, the patient died due to pneumonia. By that time, he was 84 years old.

## 3. Discussion

We found our case to be unique in medical literature. OI is highly discussed by pediatric surgeons and pediatric orthopedic surgeons [8, 9]. But only a few publications deal with fractures in adult OI patients [10, 11]. No publications have described fracture management in geriatric OI patients.

We chose an ESIN to stabilize the fracture. Due to the structure of the bone and the deformity of the femur, conventional osteosynthesis for adults would be too rigid and lead to new fractures or implant failures (Figure 1). Persiani et al. reported results with ESIN in children with type III OI and came to the conclusion that ESINs are the most suitable type of osteosynthesis for femoral shaft fractures with OI [12].

It has to be assumed that the elasticity of the femur is not only reduced because of the OI; additionally, an osteoporotic bone status must be assumed for an 83-year-old patient. Moreover, a conservative treatment from the beginning would have led to a long period of immobilization. Long-term immobilization of geriatric patients leads to a loss of independency and participation in the activities of daily life [13].

The present case shows that successful osteosynthesis with anatomical realignment is possible. The use of ESINs seems to be the method of choice, at least in this patient. Even after a second accident with a peri-implant fracture, the nail provided splinting to the new fracture and neither broke nor prevented bony healing. Of course, this is only a report of a single case, and there are limitations to any conclusions that can be drawn from our case. Nevertheless, the example shows that an ESIN is a good choice for reducing and stabilizing femoral shaft fractures in adult or even geriatric OI patients.
